# Salbutamol for analgesia in renal colic: study protocol for a prospective, randomised, placebo-controlled phase II trial (SARC)

**DOI:** 10.1186/s13063-022-06225-9

**Published:** 2022-04-25

**Authors:** Graham Johnson, Andrew Tabner, Apostolos Fakis, Rachelle Sherman, Victoria Chester, Elizabeth Bedford, Richard Jackson, Hari Ratan, Suzanne Mason

**Affiliations:** 1 Emergency Department, Royal Derby Hospital, Uttoxeter Road, Derby, DE22 3NE UK; 2 University of Nottingham, Nottingham, NG7 2RD UK; 3 Derby Clinical Trials Support Unit, Royal Derby Hospital, Uttoxeter Road, Derby, DE22 3NE UK; 4 Royal Derby Hospital, Uttoxeter Road, Derby, DE22 3NE UK; 5 Liverpool CR-UK Centre Cancer Research UK, Liverpool Cancer Trials Unit 1st Floor, C Block, Waterhouse Building, 3 Brownlow Street, Liverpool, L69 3GL UK; 6 Nottingham University Hospitals, Hucknall Rd., Nottingham, NG5 1PB UK; 7 CURE group, School of Health and Related Research, University of Sheffield, Sheffield, UK

**Keywords:** Renal colic, Kidney stones, Salbutamol, Pain, Analgesia, Emergency medicine, Urology

## Abstract

**Background:**

Renal colic is the pain experienced by a patient when a renal calculus (kidney stone) causes partial or complete obstruction of part of the renal outflow tract. The standard analgesic regimes for renal colic are often ineffective; in some studies, less than half of patients achieve complete pain relief, and a large proportion of patients require rescue analgesia within 4 h. Current analgesic regimes are also associated with significant side effects including nausea, vomiting, drowsiness and respiratory depression. It has been hypothesised that beta adrenoreceptor agonists, such as salbutamol, may reduce the pain of renal colic. They have been shown to impact a number of factors that target the physiological causes of pain in renal colic (ureteric spasm and increased peristalsis, increased pressure at the renal pelvis and prostaglandin release with inflammation). There is biological plausibility and a body of evidence sufficient to suggest that this novel treatment for the pain of renal colic should be taken to a phase II clinical trial. The aim of this trial is to test whether salbutamol is an efficacious analgesic adjunct when added to the standard analgesic regime for patients presenting to the ED with subsequently confirmed renal colic.

**Methods:**

A phase II, randomised, placebo-controlled trial will be performed in an acute NHS Trust in the East Midlands. Patients presenting to the emergency department with pain requiring IV analgesia and working diagnosis of renal colic will be randomised to receive standard analgesia ± a single intravenous injection of Salbutamol. Secondary study objectives will explore the feasibility of conducting a larger, phase III trial.

**Discussion:**

The trial will provide important information about the efficacy of salbutamol as an analgesic adjunct in renal colic. It will also guide the development of a definitive phase III trial to test the cost and clinical effectiveness of salbutamol as an analgesic adjunct in renal colic. Salbutamol benefits from widespread use across the health service for multiple indications, extensive staff familiarity and a good side effect profile; therefore, its potential use for pain relief may have significant benefits for patient care.

**Trial registration:**

ISRCTN Registry ISRCTN14552440. Registered on 22 July 2019

**Supplementary Information:**

The online version contains supplementary material available at 10.1186/s13063-022-06225-9.

## Administrative information

Note: The numbers in curly brackets in this protocol refer to SPIRIT checklist item numbers. The order of the items has been modified to group similar items (see http://www.equator-network.org/reporting-guidelines/spirit-2013-statement-defining-standard-protocol-items-for-clinical-trials/).

**Table:** 

Title {1}	Salbutamol for analgesia in renal colic: a prospective, randomised, placebo-controlled PHASE II trial (SARC)
Trial registration {2a and 2b}.	EudraCT: 2018-004305-11 ISRCTN: 14552440
Protocol version {3}	V4.0 15th July 2021
Funding {4}	The trial has been funded by the National Institute for Health Research (NIHR) Research for Patient Benefit (RfPB) grant funding scheme.
Author details {5a}	Dr. Graham Johnson^1^, Dr. Andrew Tabner^1^, Dr. Apostolos Fakis^2^, Mrs. Rachelle Sherman^2^, Professor Suzanne Mason^3,4^ ^1^REMEDY, Emergency Department, Royal Derby Hospital University Hospitals of Derby & Burton NHS Foundation Trust, Derby, DE22 3NE ^2^Derby Clinical Trials Support Unit, University Hospitals of Derby and Burton NHS Foundation Trust, Uttoxeter Road, Derby, DE22 3NE ^3^School for Health and Related Research (ScHARR), 30 Regent St, University of Sheffield, Sheffield S1 4DA ^4^Barnsley Hospital NHS Foundation Trust, Gawber Rd, Barnsley S75 2EP
Name and contact information for the trial sponsor {5b}	University Hospitals of Derby & Burton NHS Foundation Trust Research & Development Royal Derby Hospital Uttoxeter Road Derby, DE22 3NE Tel: 01332 724710 Email: uhdb.sponsor@nhs.net
Role of sponsor {5c}	The sponsor, University Hospitals of Derby and Burton NHS Foundation Trust, takes on overall responsibility for appropriate arrangements being in place to set-up, run and report the research project. The sponsor is not providing funds for this trial but has taken on responsibility for ensuring finances are in place to support the research. The sponsor has had appropriate oversight of the study design and management which has been delegated to the Derby Clinical Trials Support Unit.

## Introduction

### Background and rationale {6a}

Renal colic is the pain experienced by a patient when a renal calculus (kidney stone) causes partial or complete obstruction of part of the renal outflow tract. The lifetime incidence is approximately 12% in males and 6% in females [1] with recurrence rates approaching 50% [2]. The Royal Derby Hospital Emergency Department treats approximately 400 patients a year with renal colic.

The standard analgesic regimes for renal colic are often ineffective; in some studies, less than half of patients achieve complete pain relief, and a large proportion of patients require rescue analgesia within 4 h [3].

Current analgesic regimes are also associated with significant side effects. Treatment strategies usually involve a non-steroidal anti-inflammatory drug (NSAID) and an opiate (e.g. intravenous morphine). Opiates are known to cause nausea, vomiting, drowsiness and respiratory depression [4]. Oral absorption of NSAIDs in this cohort can be poor due to gastroparesis and vomiting; rectal administration is frequently felt by patients to be unpleasant.

The onset of action of the existing analgesic options is slow [4, 5]; NSAIDs require a period of absorption before they are effective, and intravenous opioids are controlled drugs, the administration of which is often delayed by practical concerns in their dispensing and prescription.

Our Patient and Public Involvement group has emphasised how intolerable renal colic is, how slow and inadequate the analgesic regimes can be and how unpleasant the side effects are. They have also noted the importance of remedying these factors with future research [6].

It has been hypothesised that beta adrenoreceptor agonists may reduce the pain of renal colic [7–10]. Salbutamol is a beta adrenoreceptor agonist with widespread use across the health service for multiple indications, extensive staff familiarity and a good side effect profile [11].

Beta adrenoreceptors agonists have been shown to impact a number of factors that target the physiological causes of pain in renal colic (ureteric spasm and increased peristalsis, increased pressure at the renal pelvis and prostaglandin release with inflammation) [12]. They are as follows:

- Promote ureteral relaxation [9, 13–16]
- Reduce frequency of ureteral contractions [17]
- Reduce renal pelvic pressure [18]

Approximately 60% [19] of an intravenous dose of salbutamol is excreted, unchanged, in the urine; there is therefore the potential for both systemic and local stimulations of beta adrenoreceptors to take place.

The protocol authors have completed a systematic review [20]; there have been no trials of beta agonists as analgesics in renal colic, and there are no registered clinical trials on this topic. However, there is extensive evidence (both in the laboratory and other clinical settings) suggesting it may be effective, and a number of authors have identified this as a promising research avenue.

There is biological plausibility and a body of evidence sufficient to suggest that this novel treatment for the pain of renal colic should be taken to a phase II clinical trial.

### Medical expulsive therapy and time to stone passage

Many studies have investigated agents which may decrease time to stone passage; this is not the primary outcome of interest in this trial but is included within the secondary outcomes.

It is worth noting that the previous research in this area supports the potential efficacy of salbutamol as an analgesic adjunct in renal colic via the process of ureteral relaxation (the same process thought to speed stone passage in the aforementioned studies).

The use of alpha adrenoreceptor antagonists as medical expulsive therapy to speed stone passage is a practice previously widely recommended [21] but more recently brought into question [22]. The action of alpha adrenoreceptor antagonists in the renal tract is similar to that of beta agonists; they reduce the force and frequency of ureteral contractions.

Alpha adrenoreceptor antagonists have previously been shown to reduce the number of pain episodes during the management of renal colic [21], but this has never formed the main focus of research and their use is uncommon within emergency departments in the UK; the likely reasons for this are discussed below.

#### Onset

The onset of action of salbutamol is measurable in minutes [11] whereas tamsulosin reaches peak levels after 6 h and steady state after 5 days. Salbutamol is therefore much more appropriate as a potential analgesic for acute pain in the emergency department setting.

#### Familiarity

Emergency department staff administer salbutamol in inhaled, nebulised and intravenous forms on a regular basis. This means there will be fewer barriers to adoption.

#### Side effects

The side effects of salbutamol (fast heart rate, tremor) are relatively minor compared to those of alpha blockers (low blood pressure, fainting, nausea), even at high doses, and are likely therefore to be better tolerated by patients [11, 21].

### Scientific justification

Pain in renal colic is caused first by ureteric peristalsis, followed by ureteral spasm and then subsequent inflammation and oedema [12].

β-Agonists are known to reduce ureteric peristalsis and spasm, and it is therefore hypothesised that their use will reduce the pain associated with renal colic. Additionally, salbutamol is excreted unchanged in the urine and therefore has the potential for both systemic and topical action as detailed below.

### In Vitro

- β1-, β2- and β3-adrenoceptors are found in the smooth muscle and urothelium of the human ureter [13].
- β-Agonists decrease the tone of contractions of the human ureter [13].
- Stimulation of β2 receptors decreases the contraction of the human ureter [14].
- β2 receptors are present in human ureteral smooth muscle; their stimulation mediates ureteral relaxation [15].
- A systematic review has identified that β adrenergic stimulation inhibits ureteral activity [16].

### In Vivo

- β-Agonists decrease the frequency and amplitude of contractions in the canine ureter [23].
- β-Agonists inhibit peristalsis in the canine ureter [24].
- β-Agonists reduce renal pelvic pressure and ablate ureteral peristalsis [25].
- Topical and systemic β-agonists decrease the frequency and amplitude of ureteral contractions in the pig ureter [17].

### Human evidence

- Endoluminal isoproterenol decreases renal pelvic pressure during flexible ureterorenoscopy [18, 26].
- Alpha blockers (which also mediate ureteral relaxation) have been shown to reduce the frequency of pain episodes in patients with renal colic, as well as reduce the need for other analgesics [21].

### Potential benefits

Patient and Public Involvement (PPI) work conducted by the research team demonstrated the clear and urgent need for faster, more effective pain relief that causes fewer side effects [6]. Salbutamol has the potential to fulfil that clinical and patient needs. If salbutamol is subsequently proven to be an effective analgesic in patients suffering from renal colic, the benefits are myriad:

- Improved analgesia: Pain in renal colic is caused by ureteric spasm and increased peristalsis, as well as increased pressure at the renal pelvis and prostaglandin release with inflammation [12]. Beta agonists relax the ureter, potentially providing physiologically targeted pain relief.
- Reduced time to pain relief: Salbutamol has an onset of action within 5 min, with an early peak effect [11]. This is significantly quicker than all existing analgesic options, where peak effects occur between 20 and 60 min after administration [4, 5].
- Route of administration: The need for parenteral administration was highlighted as a priority during the PPI work [6], due to the frequent association of nausea and vomiting with both renal colic and opiates. Salbutamol is solely administered parentally, and its aerosolised form means it can be administered prior to securing intravenous access, further reducing the time to analgesia.
- Better side effect profile: Salbutamol’s side effect profile is well recognised and relatively narrow, especially when compared to the combined components of the existing analgesic regime. The side effects of current treatments were also highlighted by the PPI group as a notably unpleasant part of treatment and any measures which reduce these were welcomed [6].
- The use of salbutamol may reduce the need for other analgesic agents and their associated side effects.
- Staff burden: Salbutamol is not a controlled drug, enabling nursing staff to access and administer it more readily. This reduces nursing and physician burden whilst decreasing time to administration when compared to intravenous opiates.
- Reduced admissions and length of stay: Patients with uncomplicated renal colic can be discharged once their pain is controlled; persisting pain is frequently the sole reason for continuing admission. A more effective analgesic regime may result in a shorter length of stay, whilst avoiding some admissions entirely. This has clear potential cost, service and patient benefits that will be investigated in the subsequent planned phase III trial.
- It is also possible that salbutamol may positively impact the time to stone passage.
- Home use: Patients with known renal colic may be able to self-medicate with a salbutamol inhaler, avoiding the need for hospital attendance entirely.
- Speed of adoption: Staff familiarity with salbutamol and its already widespread use means that subsequent translation into clinical practice will be easier and faster than if an alternative beta agonist were studied.

Expert advice has already been sought on the route of drug delivery from both a phase II trial methodologist (Richard Jackson, Liverpool CTU) and a Professor of Drug Discovery (Patrick Barton, University of Nottingham).

Intravenous salbutamol is the IMP for this phase II trial. Inhaled salbutamol is certainly a feasible option (and will likely form part of the phase III trial design), but for the purposes of this phase II trial, it was felt important to maximise bioavailability and reduce confounding factors in terms of absorption in order to ensure maximal safe serum levels such that any potential efficacy signal on the primary endpoint is apparent.

This trial represents a re-purposing of an established treatment. We have therefore employed the established maximum safe and efficacious intravenous dose used for acute exacerbations of asthma; this dose is safe for patients who meet the inclusion criteria [11]. A higher dose is possible but is associated with a greater frequency of side effects [11]; this dose is employed in pregnant women in pre-term labour where the potential benefits outweigh the potential harms.

The frequently occurring side effects of tremor and tachycardia are very well tolerated by the majority of patients and were felt by our patient group to be acceptable if salbutamol is proven to be an effective analgesic. Patients with ischaemic heart disease tolerate tachycardia less well and for this reason are excluded from this trial. Rare occurrences of myocardial ischaemia with the use of high doses of salbutamol have been identified [11]. The dose being administered in this trial is the typical “loading” dose of intravenous salbutamol used when patients are having an acute, severe and/or life-threatening asthma attack. Such patients have typically already received large doses of inhaled beta-agonist in addition to this intravenous dose. It is therefore not thought that the proposed dose poses a significant risk in patients without known ischaemic heart disease.

The dose and rate of administration chosen are the same as that for the relief of severe bronchospasm: 250 μg diluted to a total volume of 5 ml with 0.9% sodium chloride and given by slow intravenous injection over 3–5 min [11, 27].

Salbutamol is known to precipitate hypokalaemia [11]. The literature assessing the magnitude of this effect suggests a drop of 0.87–1.4 mmol/l [28] with a bolus dose of intravenous salbutamol. However, this trial data is largely obtained in patients with underlying hyperkalaemia, and it would appear that the lower the baseline serum potassium, the smaller the drop seen with intravenous salbutamol. Additionally, the doses used in studies identified in the review paper referenced use a higher dose of salbutamol than this trial protocol dictates. Finally, 40% of patients identified in the review paper were non-responders to salbutamol, i.e. intravenous salbutamol did not cause a fall in serum potassium. As such, we feel the potential side effect of hypokalaemia secondary to a single bolus dose of intravenous salbutamol is likely to be clinically insignificant. However, we dictate that serum potassium must be ≥ 3.7 mmol/l for a participant to be eligible for enrolment. Symptomatic hypokalaemia secondary to trial medication will be recorded as an adverse reaction.

Risks surrounding cannulation of the patient, taking of blood samples and preparation of the trial medication are covered by existing nursing staff training and procedures and provide no additional risk above normal patient care.

This trial is categorised as follows:

Type A = No higher than the risk of standard medical care—the trial involves the use of a medicinal product licenced in an EU member state, used for an off-label indication, supported by extensive clinical experience with the product and no reason to suspect a different safety profile in the trial population [29].

### Objectives {7}

The trial proposes to investigate the efficacy of salbutamol as an analgesic adjunct in patients with confirmed renal colic and to collect feasibility data to inform the development of a subsequent phase III randomised controlled trial.

#### Primary objective

To test whether salbutamol is an efficacious analgesic adjunct when added to the standard analgesic regime for patients presenting to the ED with subsequently confirmed renal colic. The addition of salbutamol will be compared to the addition of placebo to the standard analgesic regime for patients with confirmed renal colic.

#### Secondary objectives

To explore whether salbutamol could be an efficacious analgesic adjunct when added to the standard analgesic regime for patients presenting to the ED with suspected renal colic.

To assess the feasibility of conducting a definitive phase III multi-centre randomised controlled trial (RCT) of the cost and clinical effectiveness of salbutamol as an analgesic adjunct for patients with renal colic when added to the standard analgesic regime in the ED.

### Trial design {8}

This phase II randomised-controlled trial will be composed of two groups:

- Intervention group: intravenous salbutamol + standard analgesic regime
- Placebo group: intravenous sodium chloride 0.9% + standard analgesic regime

Allocation will be in the ratio of 1:1 with no stratification factors.

## Methods: participants, interventions and outcomes

### Study setting {9}

This is a single-centre study taking place in the emergency department of an acute NHS Trust in the East Midlands, UK (University Hospitals of Derby & Burton NHS Foundation Trust).

### Eligibility criteria {10}

The trial population will consist of adults (≥ 18 years old) presenting to the emergency department complaining of abdominal and/or flank pain, consistent with a working diagnosis of renal colic.

Patients aged ≥ 50 must have a serious differential diagnosis of abdominal aortic aneurysm (AAA) excluded prior to consent in line with standard practice [30]. Females of child-bearing potential must have a serious differential diagnosis of ectopic pregnancy excluded prior to consent in line with standard practice.

Potential participants will be assessed to determine the working diagnosis and immediate treatment requirements as part of routine practice. This normal treatment for patients with suspected renal colic (including standard analgesia) can be given prior to trial screening. If the working diagnosis following this assessment is felt to be renal colic, the patient will be screened for trial eligibility by one of the GCP-trained clinicians working within the department.

#### Inclusion criteria

The trial population will consist of consecutive adults presenting to the emergency department in whom all of the following apply:

1. Subjects capable of giving informed consent
2. Age ≥ 18
3. Working diagnosis of renal colic, as suggested by severe flank/unilateral abdominal pain, ± radiating to suprapubic/groin area
4. Experiencing severe pain with a requirement for intravenous analgesia, and with ongoing pain at the time of consent

#### Exclusion criteria

The participant will not enter the trial if any of the following apply:

1. Abdominal aortic aneurysm not yet excluded and participants aged ≥ 50 [30]
2. Ectopic pregnancy not yet excluded in a female of child-bearing potential
3. Currently actively taking part in another CTIMP
4. Previous participant in this trial
5. Unable to understand verbal and/or written information in English
6. Known allergy to salbutamol [11]
7. Evidence of sepsis or clinical suspicion of urinary tract infection
8. Serum potassium less than 3.7 mmol/l
9. Concomitant use of any beta blockers [11] (including beta-blocker containing eye drops) [31], prolonged-release opiates and long-acting β-agonists
10. Use of short-acting β2-agonists within the 6 h preceding presentation to the emergency department
11. Current arrhythmia (defined as non-sinus rhythm)
12. History of any of the following:

- Ischaemic heart disease
- Arrhythmogenic heart disease (not including solely patient-reported history of “palpitations”)
- Valvular heart disease
- Unilateral kidney

13. Any other contraindication to the use of salbutamol

### Who will take informed consent? {26a}

Patients will be provided with an information sheet and provided adequate time to review the information and ask any questions they may have, including discussions with the research team, non-research staff members and family and friends. Their normal treatment (standard analgesic regime) may continue independently of this decision-making time and trial screening. Whilst the PI will retain the overall responsibility for the consent of participants, they may choose to delegate the task of obtaining written consent to suitably trained medical colleagues (who have been GCP trained). Those taking consent will be required to check the eligibility of potential participants including to ensure they have sufficient capacity to consent for themselves. Informed consent must be in place prior to protocol-directed activities taking place, including any necessary screening tests.

### Additional consent provisions for collection and use of participant data and biological specimens {26b}

Consent is obtained for the use of anonymised data in future research. No further additional consent provisions are in place as no biological specimens are collected.

### Interventions

#### Explanation for the choice of comparators {6b}

Placebo has been chosen as the most appropriate comparator for this trial in order to provide a clear indication of any efficacy of salbutamol as an analgesic adjunct for renal colic. Participants are still provided standard analgesic care prior to administration of trial medication; therefore, no treatment is being withheld.

This was discussed with the PPI group during protocol development, and no concerns over the use of placebo were raised.

#### Intervention description {11a}

The intervention is a single dose of 250 μg salbutamol in 5 ml via slow intravenous injection over 3–5 min, followed by a 5-ml flush of sodium chloride 0.9%. The dose and rate of administration chosen are the same as that for the relief of severe bronchospasm [11, 27].

#### Criteria for discontinuing or modifying allocated interventions {11b}

Each participant will receive a single dose of the allocation intervention, and dose modifications are not permitted. If a participant develops clinical evidence of a significant adverse reaction during the administration of treatment, then this can be stopped at the direction of the treating clinician.

#### Strategies to improve adherence to interventions {11c}

As the intervention is a single dose administered by a healthcare professional, there are no strategies required to improve adherence. Drug accountability will be recorded on the reverse of the scratch card indicating the participant’s treatment allocation and will not be revealed to anyone except those involved in the injection preparation and designated pharmacy staff.

#### Relevant concomitant care permitted or prohibited during the trial {11d}

Concomitant medications not permitted to be taken during the patient’s participation on this trial (unless for the management of a clinical emergency, e.g. acute asthma, tachyarrhythmia) include the following:

- Any beta blockers [11] (including beta blocker-containing eye drops [31])
- Short- and long-acting β-agonists

All other concomitant medications taken by participants during their time in the study will be recorded.

#### Provisions for post-trial care {30}

As the trial involves the administration of IMP as a single dose, there is no scope for extended access to the treatment beyond the trial; therefore, continued care is not planned.

### Outcomes {12}

#### Primary endpoint/outcome

The primary outcome will be the difference in the change in pain scores (measured on a 100-mm visual analogue scale [VAS]) from baseline to 30 min post-drug administration between trial arms in patients with “confirmed renal colic”.

#### Secondary endpoints/outcomes

1. The difference in the change in pain scores (measured on a 100-mm visual analogue scale [VAS]) from baseline to 30 min post-drug administration between trial arms in patients with “suspected renal colic”
2. The difference in the change from baseline pain score to pain scores at the following time points between trial arms: 15 min, 60 min, 120 min, 240 min, and then four-hourly thereafter, until 24 h post-drug administration or hospital discharge (whichever happens first) in both of the above subgroups
3. The difference in the change in qualitative pain description from baseline pain assessment to pain assessments at the following time points between trial arms as measured using the short-form McGill Pain Questionnaire: 15 min, 30 min, 60 min and 120 min post-drug administration
4. Frequency and dose of morphine during the first 24 h from enrolment (including prehospitally)
5. Any other analgesics required and the timing of their administration
6. Length of hospital stay
7. Presence/absence, site and size of renal calculus
8. Frequency of development of acute kidney injury and date of occurrence if present
9. Degree of hydronephrosis (if present) as identified on routine imaging
10. Side effects of trial treatment
11. The mean and standard deviation of the primary outcome in participants with confirmed renal colic
12. Feasibility outcomes to inform subsequent trial design, including the following:
  - Screening rate
  - Randomisation rate
  - Recruitment rate
  - Participant retention
  - Any identified process issues
  - Volume of missing data
  - Patient compliance with trial assessments
  - Proportion of enrolled patients with confirmed renal colic
  - Emergency department diagnosis
  - Hospital discharge diagnosis
  - Patient satisfaction with the trial medication, process and delivery within the ED, including their belief regarding the arm of the trial to which they were randomised

#### Participant timeline {13}

The participant timeline is presented in Table 1.

**Table 1:** Schedule of assessments for the SARC trial

Procedures	Screening	Baseline*	Administration of trial medication									
			Time from start of trial drug administration (min)				Follow-up (h)					
			15	30	60	120	4	8	12	16	20	24
Eligibility assessment	X	X										
Demographics	X											
Informed consent	X											
ECG		X										
Potassium measurement (K^+^)		X										
Randomisation		X										
Respiratory rate Oxygen saturations Blood pressure Heart rate		X	X	X	X	X						
VAS pain score		X	X	X	X	X	X	X	X	X	X	X
McGill Questionnaire		X	X	X	X	X						
Adverse event assessments			X	X	X	X	X	X	X	X	X	X
Satisfaction Questionnaire^+^						X						
OPTIMISED SWAT Questionnaire^+^						X						
Protocol non-compliances	X	X	X	X	X	X	X	X	X	X	X	X

*All baseline assessments should take place *immediately prior* to the administration of trial treatment

^+^If the patient is discharged from the emergency department before this time point, these activities may be conducted prior to 120 min

#### Sample size {14}

This is a phase II trial to demonstrate some efficacy signal on the primary outcome. The sample size estimation has therefore been estimated based on the “probability of benefit” approach using the Mann-Whitney *U* test with the R Software [32].

Two studies [33, 34] have defined the minimum clinically significant difference between consecutive ratings of pain to be 13 mm in emergency department patients. Assuming that a difference of 13 mm between the groups in the change in pain score from baseline is clinically important (standard deviation of 20 mm—the maximum reported deviation of VAS pain at 30 min in a Cochrane Review [3], then at 5% significance level with 90% power, 53 patients with confirmed renal colic should be recruited per arm.

The standard deviation of the primary outcome in this trial will be used to inform power calculations for the subsequent definitive trial.

#### Recruitment {15}

Approximately 34 patients with a confirmed diagnosis of renal colic were discharged from the Royal Derby Hospital Emergency Department per month prior to the start of the trial. We estimate a recruitment rate of between 18 and 30% of eligible patients. This figure is derived from the current department recruitment to a comparable CTIMP (ISRCTN 34153772), another trial in an ED setting [35], and a discussion with the PPI group.

Following 11 months of study recruitment, the average number of participants with confirmed renal colic recruited in the study per month was 4. In addition, the observed proportion of participants who subsequently were found not to have a renal calculus was 30% instead of 10% that was assumed at the start of the study.

Therefore, we estimate that 106 patients with confirmed renal colic could be recruited in 31 months. This allowed for a slow start in recruitment (3 months to reach 20% recruitment rate) and recruitment plateau during the last 7 months (2 patients with confirmed renal colic recruited per month). This requires the recruitment of approximately 152 patients with suspected renal colic given that approximately 30% of patients with suspected renal colic are subsequently found not to have a renal calculus (local audit data, previous research, and first 6 months of recruitment) [36].

### Assignment of interventions: allocation

#### Sequence generation {16a}

Randomisation will be based on a computer-generated randomisation list, created using random permuted blocks of randomly varying size and implemented using a “scratch card” randomisation system. The randomisation list will be prepared using the NQuery Advisor software by an unblinded statistician. Allocation will be in the ratio of 1:1 without any stratification factors.

#### Concealment mechanism {16b}

Randomisation will be carried out using scratch cards with the allocation concealed by silver scratch-off stickers. The scratch cards will be filed in a card dispenser (or “card shoe”) to allow an unblinded staff member to draw the next card in the correct randomisation order and reduce the chance of re-ordering. The allocation is revealed by scratching off the silver area on the scratch card.

#### Implementation {16c}

An unblinded statistician will generate the randomisation list and prepare the scratch cards in order, using silver scratch-off stickers for concealing the allocation. The cards will be filed in the card dispenser and an unblinded staff member (not involved in the patient’s treatment and data collection) will draw the next card, scratch off the silver area and reveal the patient’s allocation. Patients will be enrolled and eligibility confirmed by a member of the research team before randomisation occurs.

### Assignment of interventions: blinding

#### Who will be blinded {17a}

In order to maintain the blind for treatment administration, both trial treatments will be presented as identical syringes containing 5 ml of a colourless solution labelled with a pre-printed trial label. This will be prepared by unblinded staff delegated responsibility for randomising patients and preparing the trial medication. No staff member with knowledge of the treatment allocation, will have any involvement in collecting trial data or administering trial treatment.

The SARC trial manager will be blinded to the allocation, and any monitoring that would result in unblinding of allocation will be performed by an unblended sponsor representative. Preparation of unblinded DMEC reports will be completed by a designated unblinded statistician not involved in the design and analysis of the trial.

#### Procedure for unblinding if needed {17b}

Unblinding of participants should only occur for valid medical or safety reasons, e.g. in the case of a severe adverse event where it is necessary to know which treatment the patient is receiving before they can be treated. All instances of unplanned patient unblinding should be clearly documented in the participant’s medical notes (together with the reasons for doing so) and recorded in the investigator site file. Details regarding the unblinding of participants must be forwarded to the chief investigator and the sponsor (via the Derby CTSU Trial Manager) without revealing the allocation.

The responsibility for the emergency unblinding of any participant on the trial resides with the investigator. If emergency unblinding is required for clinical reasons, this can be initiated by any treating healthcare professional. They will not be required to discuss unblinding with anyone in the research team if they feel that unblinding is necessary. The sponsor is not required to be involved in the decision to unblind a patient in an emergency situation.

The randomisation list for the trial must be held securely within the pharmacy department, in a controlled area, separate from the investigator site file and easily accessible by those authorised to reveal treatment allocation at the site.

### Data collection and management

#### Plans for assessment and collection of outcomes {18a}

Assessments will be undertaken by the staff blinded to the treatment allocation at the specified time points until either 24 h after administration of trial medication or discharge from the hospital (whichever is sooner).

All pain score measurements for the trial will use a visual analogue scale [VAS] 0–100 mm (apart from the pre-enrolment score to ensure eligibility, which will be a NAS as per routine practice). The visual analogue scale will be a 100-mm line with cues at either end (0 = no pain, 100 mm = worst pain), and participants will be asked to mark their current pain score with a cross at each time point listed below. The qualitative assessment of participants’ pain will be obtained using the short-form McGill Pain Questionnaire.

Participants will be asked to complete a satisfaction questionnaire regarding pain relief and their experience of the trial in the emergency department. The presence/absence of renal calculus will be determined by appropriate imaging (CT renal tract or XR KUB); this takes place during normal treatment and within 24 h of admission in routine practice. The research team will record trial-specific observations directly onto the eCRF.

Information about AKI development within 7 days from admission will be collected from the patient’s notes retrospectively.

#### Plans to promote participant retention and complete follow-up {18b}

Participant dropout/loss to follow-up is not expected to present an issue due to the short duration of patient involvement; however, it is recognised that the frequency of assessments may result in a higher incidence of missing data. Participants will be actively monitored for safety reasons until 120 min post-treatment administration and therefore the research staff should be able to ensure the completion of outcome data. Should the participant be transferred to a different department, they will be provided with a booklet containing the VAS pain score outcomes and asked to complete these. A timer will be provided to serve as a reminder for when assessments are needed.

### Data management {19}

#### Data collection tools and source document identification

An electronic software platform will be used for trial data capture. Data capture will be via a web-based, fully validated system, compliant with 21 CRF Part 11; Electronic records; Electronic signatures and EU Commission Directive 2005/28/EC with comprehensive audit trials. DCTSU will be responsible for database build and system validation. Data will be hosted externally according to General Data Protection Regulation guidance.

#### Source data

Source data will consist of paper and electronic medical records depending on the data being collected. Patient-reported outcomes (specifically the McGill Questionnaire, Patient Satisfaction and VAS) will be recorded directly onto paper which will serve as the source data prior to being transcribed onto the eCRF. There may be some instances where the data is transcribed directly onto the eCRF, and this will be determined with the PI prior to the start of the trial at the site.

#### Data handling and record keeping

The investigator and trial team will ensure that the participant’s identity is protected at every stage of their participation within the trial, according to the Caldicott principles. If any patient information needs to be sent to a third party, the trial team will adhere to maintaining pseudo-anonymous participant parameters in correspondence.

The trial database will be designed to capture the clinical data in accordance with the best principles of clinical data management and the relevant SOPs on Clinical Data Management System Specification and Validation, Data Capture, Instrument Design and Database Development developed by the Derby CTSU.

Access to the trial database will be restricted by role-based permission to authorised trial personnel. Users will be suitably trained on the system prior to being granted access. Individual user accounts will be password-protected and will not be shared between members of the trial team.

Data will be entered into the eCRF using worksheets and source documents at the site. Post-data entry, validation checks will be performed on the data to ensure accuracy and consistency according to the data validation plan. All data queries generated as a result of these checks will be available for resolution by the site online. After data entry is complete, all data queries have been resolved and medical coding is completed, the database will be locked and released for statistical analysis.

All clinical data will be collected, stored, processed and archived in accordance with the Data Management Plan for this trial and in line with the relevant SOPs on data entry, data quality assessment, data validation, database lock and data transfer and archiving developed by the Derby CTSU and any relevant legislation.

Access will be granted to authorised representatives from the sponsor, trial team and the regulatory authorities to permit trial-related monitoring, audits and inspections. The purpose of these inspections is to verify and corroborate the data collected on the case report forms. In order to do this direct access to medical or clinic records is necessary. The CI/PI must inform the sponsor if they are notified of a forthcoming audit by the IEC/IRB or regulatory authorities.

The principal investigator will ensure that the following information is contained in the medical or clinic records of the participant and that the entries are signed and dated:

- Sufficient data to allow verification of the entry criteria in terms of past and present medical and medication histories.
- The day the participant entered the trial describing the trial number, the treatment being evaluated, the unique number assigned to the participant and a statement that informed consent was obtained.
- Each subsequent trial visit including any concerns about adverse events and their resolution.
- Any deviation from the protocol procedures and subsequent impact on endpoint data validity.
- All concomitant medication taken by the participant, including start and stop dates.
- The date when the participant finished the trial, the reason for termination and the participant’s general condition at trial completion.

#### Access to data

Direct access will be granted to authorised representatives from the sponsor, Derby CTSU, host institution and regulatory authorities to permit trial-related monitoring, audits and inspections.

#### Confidentiality {27}

The trial will be conducted in accordance with the Data Protection Act 2018 and other applicable legislation, including but not limited to the EU General Data Protection Regulation. The investigator must ensure that the participant’s anonymity is maintained throughout the trial and following completion of the trial. Participants will be identified on all trial-specific documents (except for the screening log, informed consent form and enrolment log) only by the participants’ trial-specific identifier. This identifier will be recorded on all trial documents and the database. The investigator site file will hold an identification log detailing the trial-specific identifier alongside the names of all participants enrolled in the trial.

All documents will be stored securely with access restricted to trial staff and authorised personnel.

Dr Graham Johnson, as the chief investigator, will act as the custodian of the data generated in the trial.

#### Plans for collection, laboratory evaluation and storage of biological specimens for genetic or molecular analysis in this trial/future use {33}

The study does not collect biological specimens for analysis and storage or for use in future research.

## Statistical methods

### Statistical methods for primary and secondary outcomes {20a}

The statistical analysis will be undertaken by the trial statistician. The trial statistician will draft the statistical analysis plan (SAP), which will be reviewed by the Trial Management Group (TMG), the Trial Steering Committee (TSC) and the Data Monitoring and Ethics Committee (DMEC). The finalised SAP will be approved and signed by the CI and the trial statistician.

### Primary outcome analysis

The primary outcome of the change in pain scores (measured with VAS) from baseline to 30 min in patients with “confirmed renal colic” will be compared between the two trial arms using the Mann-Whitney *U* test. Further analysis of the primary endpoint will be carried out using an analysis of covariance (ANCOVA) approach, analysing the pain scores at 30 min and including the baseline pain scores as a covariate, along with any other clinical/demographic covariates of import, e.g. age, gender and weight. The results of the primary endpoint will be reported as the mean change in pain score for each treatment arm along with associated 95% confidence intervals.

### Secondary outcome analysis

#### Pain scores

The secondary outcome of the change in pain scores (measured with VAS) from baseline to 30 min in patients with “suspected renal colic” will be compared between the two trial arms using the Mann-Whitney *U* test. The change in pain scores (measured with VAS) from baseline to 15, 60, 120 and 240 min and four-hourly thereafter in patients with “confirmed renal colic” and with “suspected renal colic” will be compared between the two trial arms at each time point using the Mann-Whitney *U* test, and across all time points using repeated measures ANCOVA including the baseline pain scores as a covariate, along with any other clinical/demographic covariates of import.

The change in pain scores (measured with the McGill Pain Questionnaire) from baseline to 15, 30, 60 and 120 min in patients with “confirmed renal colic” and with “suspected renal colic” will be compared between the two trial arms at each time point using the Mann-Whitney *U* test and across all time points using repeated measures ANCOVA including the baseline pain scores as a covariate, along with any other clinical/demographic covariates of import.

#### Clinical outcomes

Secondary continuous outcomes (length of stay, degree of hydronephrosis) will be compared between the two treatment groups using the Mann-Whitney *U* test. Secondary categorical outcomes (frequency and dose of morphine, other analgesics required, and presence, site and size of renal calculus) will be compared between the two treatment groups using the chi-squared test.

#### Feasibility outcomes

Descriptive statistics will be presented to summarise the feasibility outcomes across each of the randomisation groups, where relevant. The continuous feasibility outcomes will be reported with medians and interquartile ranges (IQR), whilst the categorical feasibility outcomes will be reported with frequencies and percentages.

#### Patient Satisfaction Questionnaire

Frequencies and percentages will be used to report the responses in the patient satisfaction questionnaire by treatment group and will be compared using a chi-square test.

#### Toxicity

The number and percentage of patients reporting a SAE or SUSAR will be summarised by treatment group and compared using a chi-square test.

### Interim analyses {21b}

No formal interim analysis is planned, but the sponsor and funder reserve the right to discontinue this trial at any time for ethical, safety or any other administrative reason. If this occurs ,the sponsor shall justify its decision in writing and will promptly inform any relevant parties (i.e. investigators, participating sites, REC, regulatory bodies).

The sponsor and funder shall take advice from the Trial Steering Committee as appropriate in making this decision. An independent Data Monitoring and Ethics Committee shall monitor accumulating data and oversee safety issues. The reporting requirements and frequency of reports will be defined in the TSC and DMEC Charters.

The DMEC will advise the TSC if, in its view, there are any ethical or safety issues that may necessitate the closure of the trial. These issues include (but are not limited to) the following:

- Prevalence of excess side effects, SARs or SUSARs in the intervention group deemed unacceptable as defined by the DMEC

### Methods for additional analyses (e.g. subgroup analyses) {20b}

At randomisation, the final diagnosis of the recruited participants is unknown, and hence, we randomise all participants with “suspected renal colic”. However, this is a phase II efficacy trial, and the primary group of interest is the patients with “confirmed renal colic”, which is a diagnosis that we know at participant’s discharge. Therefore, the primary analysis of the primary endpoint will be carried out within the “confirmed renal colic” group on the full data set, which will be defined on the “modified” intention-to-treat principle retaining patients in their initially randomised groups irrespective of any protocol violations. Analyses of the “suspected renal colic” and the “other diagnosis” groups for all secondary endpoints will also be done on the “modified” intention-to-treat principle.

Secondary analysis of the primary endpoint will be carried out within the “confirmed renal colic” and “suspected renal colic” groups on the per-protocol principle by excluding any patients with major protocol deviations. In addition, the analysis of the primary endpoint will be undertaken on the “as treated” principle by including patients in the treatment group of the actual medication they have received.

Analysis of harms (adverse events) will be restricted to participants who received the allocated trial medication, so that absence or occurrence of harm is not attributed to a treatment that was never received.

### Methods in analysis to handle protocol non-adherence and any statistical methods to handle missing data {20c}

Missing data are expected to be small and final analyses are planned to be carried out on a complete case basis; any participant in whom the imaging necessary to obtain specific secondary outcome data (e.g. degree of hydronephrosis) is not performed will be excluded from that portion of the data analysis.

If there is missing data in the primary endpoint, then multiple imputation using chained equations will also be applied. If substantial missing data (> 10%) are observed in either a secondary trial outcome or key prognostic covariate, then multiple imputation using chained equations will be applied.

### Plans to give access to the full protocol, participant-level data and statistical code {31c}

As an investigator-led trial, access to the final trial dataset will be restricted to the CI, the trial statistician and the appropriate members of Derby Clinical Trials Support Unit and the sponsor. External investigators will be required to submit a formal request to the Trial Management Group for access to data.

### Study within a trial

The trial serves as a host trial for a Study Within A Trial (SWAT) to assess the impact of different participant information sheets on recruitment rates. Patients identified as eligible to take part in the main trial will be provided with either PIS A (optimised format, an A4 booklet) or PIS B (conventional format). This will be determined randomly and patients will not be made aware of the different formats available.

Participants should also be asked to complete the optional “OPTIMISED Decision-Making Questionnaire” at the 120-min follow-up (or on emergency department discharge) that will assess patient satisfaction with the participant information sheet they were given. The SWAT is registered as SWAT 101 on the MRC Hub for Trials Methodology SWAT Repository Stored (ref:https://www.qub.ac.uk/sites/TheNorthernIrelandNetworkforTrialsMethodologyResearch/SWATSWARInformation/Repositories/SWATStore/).

### Oversight and monitoring

#### Composition of the coordinating centre and trial steering committee {5d}

University Hospitals of Derby and Burton NHS Foundation Trust (UHDB), as the sponsor of this trial, has delegated certain duties to the Derby Clinical Trials Support Unit and the chief investigator in the conduct of the trial, as outlined in a tripartite Division of Responsibilities. UHDB controls the final decision regarding any aspects of the trial, as outlined within this tripartite agreement.

#### Trial management group

The trial management group will meet regularly (as detailed within the trial monitoring plan) to oversee the day-to-day management of the trial, including all aspects of the conduct of the trial. Any problems with trial conduct and participating centres will be raised and addressed during TMG meetings.

#### Trial steering committee

The trial steering committee will oversee and supervise the progress of the trial and ensure that it is being conducted according to ICH-GCP and the applicable regulations. The TSC is an independent body that includes majority members who are not involved with the running of the trial (known as independent members). Membership includes clinicians with trial expertise, a statistician and a PPI representative.

TSC meetings will be held according to the monitoring plan and may be conducted in person or remotely via teleconference.

#### Composition of the data monitoring committee, its role and reporting structure {21a}

The data monitoring and ethics committee will review the accruing trial data and will assess whether there are any safety issues that should be brought to the participant’s attention or any reasons to terminate the trial. They will also review the scientific validity and the conduct of the trial. DMEC meetings will be held according to the monitoring plan. The DMEC is fully independent and consists of a statistician and two clinicians with trial expertise.

#### Adverse event reporting and harms {22}

The use of salbutamol is outside of its licenced indication, but with a well-known safety profile and no reason to suspect a change in the safety profile for the population of patients included in the trial. For this trial, it is expected that all adverse events (AEs) that show a potential causal relationship with the IMP, known as adverse reactions (ARs), are recorded. Other AEs of unexpected severity (in the opinion of the investigator), or which meet the criteria for a serious adverse event (SAE) should also be recorded. They should be recorded using the CTCAE term provided in the NCI CTCAE v5.0. Severity should be assessed using the NCI CTCAE v5.0 grading. The clinical course of each event should be followed until resolution or stabilisation.

Events that are recognised, and expected complications of renal colic are not required to be reported as adverse events unless they are of an unexpected severity (i.e. require an intervention not usually required in the management of renal colic and its complications), are thought to be related to the IMP (and are therefore ARs) or meet the definition of serious.

The following circumstances are usually not considered SAEs:

- Routine treatment or monitoring of the studied indication not associated with any deterioration in condition
- Treatment which was elective or pre-planned for a pre-existing condition not associated with any deterioration in condition
- Any admission to a hospital or other institution for general care where there was no deterioration in condition
- Treatment of an emergency on an outpatient basis for an event not fulfilling any of the definitions of serious as given above and not resulting in hospital admission

All AE/ARs and SAE/SARs (not considered exempt) must be recorded from the time of trial medication administration until the end of the participant’s last data collection. Due to the fast-acting nature and short half-life of salbutamol, active monitoring for AEs and ARs is not required after 2 h post-administration. Following this, investigators are still required to record any ARs or SARs they become aware of.

Each SAE that is assigned as both suspected to be related to IMP treatment and unexpected will be initially classified as a SUSAR and reported to the sponsor who will take necessary steps to reveal the treatment allocation of the individual participant concerned and report to the MHRA if required within the required expedited reporting timescales.

Safety information will be reviewed for ongoing assessment of the risk/benefit during Data Monitoring and Ethics Committee (DMEC) meetings.

#### Frequency and plans for auditing trial conduct {23}

Authorised representatives of the sponsor and competent authority may visit the participating sites to conduct independent audits/inspections according to a pre-determined audit plan.

Monitoring and source data verification will be conducted by the Derby CTSU according to the trial monitoring plan. The extent and nature of monitoring will be determined by the trial objectives, purpose, design, complexity, blinding, number of patients and sites and endpoints.

#### Plans for communicating important protocol amendments to relevant parties (e.g. trial participants, ethical committees) {25}

Changes to the protocol will be documented in written protocol amendments; the Derby CTSU is responsible for deciding if an amendment should be deemed substantial or non-substantial. Substantial amendments will be submitted to the relevant regulatory bodies (MHRA, REC, HRA) for review and approval. The amendments will only be implemented after approval and a favourable opinion has been obtained. Non-substantial amendments will be submitted to the HRA for their approval/acknowledgement. Amendments will not be implemented until all relevant approvals are in place, including local site approval. As participants are in the trial for up to 24 h, it is not expected that participants will be asked to re-consent as a result of any protocol amendments.

#### Dissemination plans {31a}

Upon completion of the trial, an end of trial report will be generated and submitted to REC within 12 months of the end of trial. As the funder for the trial, the NIHR will also be provided with a report of the trial, per their requirements.

The results of this trial will be submitted to peer-reviewed journals for publication as soon as data analysis is completed. Participants will not be identified in any publications. The PPI representatives involved in the trial will support the dissemination of the information into the public domain and to the participants involved in the trial, in an appropriate manner.

##### Conference proceedings

The findings will be presented at national and international emergency medicine and urology conferences, e.g. the Royal College of Emergency Medicine Annual Scientific Conference and Clinical Studies Group meetings, and the British Association of Urological Surgeons Endourology meeting.

##### Online

The findings will be presented in online fora including podcasts and blogs, e.g. RCEMLearning FOAM Network.

##### Social media

The findings will be disseminated and publicised through links with organisations with a large social media presence.

## Discussion

The study remains ongoing but offered some logistical challenges identified during set-up which were addressed by the team.

- The study’s limited funding meant that pre-filled syringes could not be provided for the team therefore a different strategy to maintain the blind was required. As the trial medication is made up in a department, and not by a separate pharmacy, non-research team members are required to make up either the IMP or placebo and provide in a blinded syringe for the research team to administer. Designated members of a separate research team, not working on this trial, will be unblinded and support the SARC research team and R&D Pharmacy team in monitoring drug accountability and re-ordering stock for the department. No one with knowledge of a participant’s allocation will be involved in data collection or patient monitoring for the trial.
- Further to this, as non-research staff members will be randomising patients, a simple randomisation system was required that avoided the need for an additional log in or access to a computer as is the case with online randomisation systems. Building on work done in the Rapid Analgesia for Prehospital Hip Disruption (RAPID) study (ref) using scratch cards for concealment allocation, we decided to implement this as a simple method of randomisation for the SARC trial. We will monitor randomisation adherence and intend to report back on this alongside user acceptability in a separate study.

## Trial status

The trial began recruitment on 16th September 2019 and is anticipated to complete recruitment on 31st October 2022. Recruitment was paused in March 2020 due to the COVID-19 pandemic and recommenced on 4th November 2020. An extension to the trial has been granted to take account of this pause and the slower than anticipated recruitment. The current protocol version is v4.0 dated 15th July 2021.

## Supplementary Information

**Additional file 1.**
