# Supplementary material for: Salbutamol for analgesia in renal colic: study protocol for a prospective, randomised, placebo-controlled phase II trial (SARC)
Source: Trials. 2022 Apr 25;23:352. doi: 10.1186/s13063-022-06225-9 (PMC9036510; doi:10.1186/s13063-022-06225-9)
Supplement: Supplementary file 1 — Additional file 1. [file 13063_2022_6225_MOESM1_ESM.docx]

**Statistical Analysis Plan**

**Salbutamol for Analgesia in Renal Colic: A prospective, randomised, placebo-controlled Phase II trial (SARC)**

|  | **Name** | **Signature** | **Date**  ***(DD/MMM/YYYY)*** |
| --- | --- | --- | --- |
| **Chief Investigator** | Dr Graham Johnson | 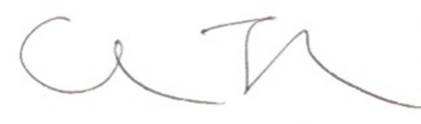 | 15/11/2021 |
| **Study Statistician** | Dr Apostolos Fakis |  |  |

| **Protocol Version Number and Date** | 4.0, 02/JUL/2021 | **IRAS reference** | *252075* |
| --- | --- | --- | --- |

| **Version Number** | **Date** | **Author** | **Description of Changes** |
| --- | --- | --- | --- |
| 1.0 | 12/DEC/2019 | Apostolos Fakis | Original version. |
| 2.0 | 28/APR/2021 | Apostolos Fakis | Incorporated the DMEC suggestions. |
| 3.0 | 02/NOV/2021 | Apostolos Fakis | Incorporated the DMEC suggestions. |

Table of Contents

[[A] Sample Size 3](#_Toc27034902)

[[A.1] Sample Size Estimation / Power Calculation [Protocol Sec. 11.1 & 11.2] 3](#_Toc27034903)

[[A.2] Sample Size Amendments After Interim Analysis 3](#_Toc27034904)

[[A.3] Final Sample Size 3](#_Toc27034905)

[[B] Randomisation [Protocol Sec. 7.4] 3](#_Toc27034906)

[[C] Interim Analysis [Protocol Sec. 11.6] 3](#_Toc27034907)

[[C.1] Justification for Interim Analysis 3](#_Toc27034908)

[[C.2] Definition of Endpoints used in Interim Analysis 3](#_Toc27034909)

[[C.3] Statistical Methods for Interim Analysis 3](#_Toc27034910)

[[D] Final Statistical Analysis 4](#_Toc27034911)

[[D.1] Summary of Baseline Data [Protocol Sec. 11.3.1] 4](#_Toc27034912)

[[D.2] Definition of Primary Endpoint [Protocol Sec. 3.3] 4](#_Toc27034913)

[[D.3] Statistical Methods for Primary Analysis [Protocol Sec. 11.3.2] 4](#_Toc27034914)

[[D.4] Definition of Secondary Endpoints [Protocol Sec. 3.4] 4](#_Toc27034915)

[[D.5] Statistical Methods for Secondary Analyses [Protocol Sec. 11.3.3] 5](#_Toc27034916)

[[D.6] Statistical Methods for Sub-group Analyses [Protocol Sec. 11.4] 5](#_Toc27034917)

[[D.7] Statistical Methods for Sensitivity Analyses [Protocol Sec. 11.5] 6](#_Toc27034918)

[[D.8] Definition of Safety Endpoints 6](#_Toc27034919)

[[D.9] Statistical Methods for Safety Endpoints [Protocol Sec. 11.3.3] 6](#_Toc27034920)

[[E] Analysis Groups and Missing Data 6](#_Toc27034921)

[[E.1] Definition of Analysis Groups [Protocol Sec. 11.7] 6](#_Toc27034922)

[[E.2] Definition of Inclusion Groups [Protocol Sec. 11.8] 6](#_Toc27034923)

[[E.3] Procedure for Accounting for Missing, Unused, and Spurious Data [Protocol Sec. 11.8] 7](#_Toc27034924)

[[F] Unplanned Analyses 7](#_Toc27034925)

[[F.1] Unplanned Analyses Requested by the CI 7](#_Toc27034926)

[[F.2] Unplanned Analyses Requested by the Sponsor 7](#_Toc27034927)

[[F.3] Unplanned Analyses Requested by the Journal Reviewer 7](#_Toc27034928)

[[G] Comments 7](#_Toc27034929)

[Appendix A. Dummy Tables Error! Bookmark not defined.](#_Toc27034930)

[Appendix B. CONSORT Diagram Error! Bookmark not defined.](#_Toc27034931)

# Sample Size

## Sample Size Estimation / Power Calculation [Protocol Sec. 11.1 & 11.2]

- - 1. The sample size estimations and planned recruitment rate are described in the current protocol.

## Sample Size Amendments After Interim Analysis

- - 1. The conversion rate of patients with suspected renal colic to confirmed renal colic was reviewed in April 2021. While the original conversion rate was 90%, the actual one observed in the trial was 70%. Therefore, the number of patients with suspected renal colic has been adjusted to 152, while the number of patients with confirmed renal colic remained the same at 106.

## Final Sample Size

- - 1. Approximately 152 patients with **suspected** renal colic so that to achieve 106 with **confirmed** renal colic.

# Randomisation [Protocol Sec. 7.4]

- - 1. The randomisation method and process are described in the current protocol and in the randomisation specification (v1.2 for full trial and v1.0 for SWAT).

# Interim Analysis [Protocol Sec. 11.6]

## Justification for Interim Analysis

- - 1. Interim analysis by treatment group will only be done as part of the unblinded report for the Data and Monitoring Ethics Committee (DMEC). There will not be any formal analyses presented which will impact on the error rates of the study (e.g. alpha level).

## Definition of Endpoints used in Interim Analysis

- - 1. The endpoints used in the unblinded DMEC reports are defined in the DMEC Charter and the “SARC DMEC Dummy Tables” document.

## Statistical Methods for Interim Analysis [Protocol Sec. 11.6]

- - 1. Descriptive statistics will be presented to summarise the distribution of baseline variables across each of the randomisation groups. The continuous baseline variables (e.g. age, weight and size of stone) will be reported with means (SD), and medians [Interquartile Ranges (IQR)]. Categorical variables (gender, presence / site of stone, hydronephrosis, AKI on presentation) will be reported with frequencies and percentages.
    2. The primary outcome of the change in pain scores (measured with VAS) from baseline to 30 minutes in patients with **“Confirmed Renal Colic”** will be compared between the two groups using Mann U Whitney test. Results will be reported as the median change in pain score for each treatment arm along with the difference in median changes between the two groups and 95% confidence intervals using the Bonett-Price method.
    3. The secondary outcome of the change in pain scores from baseline to 30 minutes, and the length of hospital stay in patients with **“Suspected Renal Colic”** will be compared between the two groups using Mann U Whitney test. The difference in medians (95% CIs) will be presented. Use of analgesics and presence of AKI within 7 days from admission will be compared between the two treatment groups using Chi-squared test. Ratio of rates (95% CIs) will be presented.
    4. Frequency (%) will be presented per treatment group for non-compliances, AEs, SAEs and SUSARs. A list with all AEs, SAEs and SUSARs per group will be presented.

# Final Statistical Analysis

## Summary of Baseline Data [Protocol Sec. 11.3.1]

- - 1. Descriptive statistics will be presented to summarise the distribution of baseline variables across each of the randomisation groups. The continuous baseline variables (e.g. age, weight and size of stone) will be reported with medians (IQR). Categorical variables (gender, presence / site of stone, hydronephrosis, AKI on presentation) will be reported with frequencies and percentages.
    2. A Consolidated Standards of Reporting Trials (CONSORT) flow diagram will be produced, showing the frequency of participants:
- Screened, and reasons for not eligible,
- Eligible, and reasons for exclusion,
- Provided consent,
- Excluded before randomisation and the reason for exclusion,
- Randomised,
- Allocated to each randomisation group,
- That received each allocated intervention,
- That did not receive each allocated intervention,
- Not completed the study, and the frequency of each reason per randomisation group,
- Per analysis group,
- Not analysed (and the frequency of each reason for not being analysed) for each randomisation group /sub-group.

## Definition of Primary Endpoint [Protocol Sec. 3.3]

- - 1. The primary endpoint is the difference in the change in pain scores (measured on an 100mm Visual Analogue Scale [VAS]) from baseline to 30 minutes post drug administration between trial arms in patients with "**Confirmed** Renal Colic".

## Statistical Methods for Primary Analysis [Protocol Sec. 11.3.2]

- - 1. The primary outcome of the change in pain scores (measured with VAS) from baseline to 30 minutes in patients with **“Confirmed Renal Colic”** will be compared between the two trial groups using Mann U Whitney test. Results will be reported as the median change in pain score for each treatment group along with the difference in median changes between the two groups and 95% confidence intervals using the Bonett-Price method.
    2. Further analysis of the primary endpoint will be carried out using an Analysis of Covariance (ANCOVA) approach, analysing the pain scores at 30 minutes and including the baseline pain scores as a covariate, along with any other clinical/demographic covariates of import. Results will be reported as the mean change in pain score for each treatment group along with the difference in mean changes between the two groups and their associated 95% confidence intervals.
    3. Analysis of the primary outcome will be assessed using 2-sided 0.05 level, as is consistent with the type I alpha level used in the trial design.

## Definition of Secondary Endpoints [Protocol Sec. 3.4]

- - 1. The secondary endpoints are defined in the current protocol.

## Statistical Methods for Secondary Analyses [Protocol Sec. 11.3.3]

- - 1. Pain Scores: The secondary outcome of the change in pain scores (measured with **VAS**) from baseline to 30 minutes in patients with **“Suspected Renal Colic”** will be compared between the two groups using Mann U Whitney test.
    2. Pain Scores: The change in pain scores (measured with **VAS**) from baseline to 15, 60, 120, 240 minutes, and four-hourly thereafter in patients with “Confirmed Renal Colic” and with “Suspected Renal Colic” will be compared between the two trial arms at each time point using Mann Whitney U test, and across all time points using repeated measures ANCOVA including the baseline pain scores as a covariate, along with any other clinical / demographic covariates of import, e.g. age, gender, and weight.
    3. Pain Scores: The change in pain scores (measured with **McGill Pain Questionnaire**) from baseline to 15, 30, 60, and 120 minutes in patients with “Confirmed Renal Colic” and with “Suspected Renal Colic” will be compared between the two groups at each time point using Mann Whitney U test, and across all time points using repeated measures ANCOVA including the baseline pain scores as a covariate, along with any other clinical/demographic covariates of import, e.g. age, gender, and weight.
    4. Clinical Outcomes: Secondary continuous outcomes (length of stay, degree of hydronephrosis) will be compared between the two treatment groups using Mann Whitney U test.
    5. Clinical Outcomes: Secondary categorical outcomes (timing, frequency of morphine prior and after the trial treatment, other analgesics required, presence of AKI within 7 days from admission) will be compared between the two treatment groups using Chi-squared test/Fishers test as appropriate for the categorical variables and Mann Whitney U test for the continuous.
    6. Feasibility Outcomes: Descriptive statistics will be presented to summarise the feasibility outcomes (e.g. rate of screening/recruitment, completeness of data, patient retention) across each of the randomisation groups, where relevant.
    7. Patient Satisfaction Questionnaire: Frequencies and percentages will be used to report the responses in the patient satisfaction questionnaire by treatment group and will be compared using a Chi-Square test.
    8. Results from Mann Whitney U test will be reported as the difference in medians between the two groups and their associated 95% confidence intervals using the Bonett-Price method. Results from the ANCOVA will be reported as the difference in means between the two groups and their associated 95% confidence intervals. Results from Chi-Squared test will be reported as the difference in proportions and ratio of proportions with their associated 95% confidence intervals.

## Statistical Methods for Sub-group Analyses [Protocol Sec. 11.4]

- - 1. Analyses including all patients categorised as **“other diagnosis”** will be undertaken for the primary outcome using Mann U Whitney test. Results will be reported as the median change in pain score for each treatment group along with the difference in median changes between the two groups and 95% confidence intervals using the Bonett-Price method.
    2. The trial population will be divided to pre and post COVID19 groups (“pre” will be those recruited prior to lockdown, and “post” those recruited following the restart of the study after lockdown). Descriptive statistics will be presented to summarise the distribution of baseline variables across each of the COVID19 groups. Analysis of Covariance (ANCOVA) approach will also be undertaken analysing the pain scores at 30 minutes and including the baseline pain scores and an interaction term of treatment group by COVID19 group as covariates, along with any other clinical/demographic covariates of import, e.g. age, gender, and weight.

## Statistical Methods for Sensitivity Analyses [Protocol Sec. 11.5]

- - 1. Further analysis of pain scores over time will be examined using linear mixed models including treatment by time interaction, and patient ID as a random effect to ensure both within patient and between patient levels of variability are estimated. Normality of model residuals, where assumed, will be assessed using residual plots and appropriate data transformations applied where necessary.
    2. Further sensitivity analysis based on the “per protocol” and the “as treated” analysis sets will be undertaken only for the primary endpoint for both the “Confirmed Renal Colic” and “Suspected Renal Colic” groups.
    3. If missing data are observed in the primary or secondary endpoints, then sensitivity analysis will be performed as described in section E.3.1.

## Definition of Safety Endpoints

- - 1. The safety endpoints will be the SAEs and SUSARs reported.

## Statistical Methods for Safety Endpoints [Protocol Sec. 11.3.3]

- - 1. Toxicity: The number and percentage of patients reporting a SAE or SUSAR will be summarised by treatment group and compared using a Chi-Square test.

# Analysis Groups and Missing Data

## Definition of Analysis Groups [Protocol Sec. 11.7]

- - 1. **Modified Intention to Treat (modified ITT)**: At randomisation, the final diagnosis of the recruited participants is unknown, and hence we randomise all participants with “Suspected Renal Colic”. However, this is a phase II efficacy trial, and the primary group of interest are the patients with “Confirmed Renal Colic”, which is a diagnosis that we know at participant’s discharge. Therefore, the primary analysis of the primary endpoint will be carried out within the “**Confirmed** Renal Colic” group on the full data set, which will be defined on the “modified intention to treat” principle **retaining patients in their initially randomised groups irrespective of any protocol violations**. Analyses of the “**Suspected** Renal Colic” and the “**Other Diagnosis**” groups for all secondary endpoints will also be done on the “modified” intention to treat principle.
    2. **Per Protocol**: Secondary analysis of the primary endpoint will be carried out within the “Confirmed Renal Colic” and “Suspected Renal Colic” groups on the “per protocol” principle by **excluding any patients with major protocol deviations**.
    3. **As Treated**: Analysis of the primary endpoint will be undertaken on the “as treated” principle by **including patients in the treatment group of the actual medication they have received**.
    4. Analysis of harms (adverse events) will be restricted to participants who received the allocated trial medication, so that absence or occurrence of harm is not attributed to a treatment that was never received.

## Definition of Inclusion Groups [Protocol Sec. 11.8]

- - 1. Complete Dataset Population: Missing data are expected to be small and final analyses are planned to be carried out on a complete case basis; any participant in whom the imaging necessary to obtain specific secondary outcome data (e.g. degree of hydronephrosis) is not performed will be excluded from that portion of the data analysis.

## Procedure for Accounting for Missing, Unused, and Spurious Data [Protocol Sec. 11.8]

- - 1. If there is missing data in the primary endpoint, then multiple imputation using chained equations will also be applied. If substantial missing data (>10%) are observed in either a secondary trial outcome or key prognostic covariate, then multiple imputation using chained equations will be applied.

# Unplanned Analyses

## Unplanned Analyses Requested by the CI

## Unplanned Analyses Requested by the Sponsor

## Unplanned Analyses Requested by the Journal Reviewer

# Comments

- - 1. *The statistical analysis plan has been reviewed by Graham Johnson, Andrew Tabner and Richard Jackson.*
    2. *All statistical analyses will be conducted using Stata software.*
